# Outcomes in small children on Berlin Heart EXCOR support: age and body surface area as clinical predictive factors

**DOI:** 10.1093/ejcts/ezac516

**Published:** 2022-11-08

**Authors:** Sofie Rohde, Eugen Sandica, Kevin Veen, Ulrike S Kraemer, Timothy Thiruchelvam, Oliver Miera, Maria L Polo Lopez, Joanna Sliwka, Antonio Amodeo, Ad J J C Bogers, Theo M M H de By

**Affiliations:** Department of Cardio-Thoracic Surgery, Erasmus University Medical Center, Rotterdam, Netherlands; Clinic for Pediatric Cardiac Surgery and Congenital Heart Defects, Heart and Diabetes Centre North Rhine-Westphalia, Ruhr-University of Bochum, Bad Oeynhausen, Germany; Department of Cardio-Thoracic Surgery, Erasmus University Medical Center, Rotterdam, Netherlands; Department of Paediatric Intensive Care, Erasmus University Medical Center, Rotterdam, Netherlands; Great Ormond Street Hospital, London, UK; Department of Congenital Heart Disease and Pediatric Cardiology, Deutsches Herzzentrum Berlin, Berlin, Germany; Department of Pediatric and Congenital Cardiac Surgery, La Paz University Hospital, Madrid, Spain; Department of Cardiac Surgery, Transplantology and Vascular Surgery, Silesian Center for Heart Diseases, Zabrze, Poland; Ospedale Bambino Gesù, Rome, Italy; Department of Cardio-Thoracic Surgery, Erasmus University Medical Center, Rotterdam, Netherlands; EUROMACS, EACTS House, Windsor, UK

**Keywords:** Berlin Heart EXCOR, Ventricular assist device, Paediatric, Mortality, VAD explantation, Heart transplantation, EUROMACS

## Abstract

**OBJECTIVES:**

The Berlin Heart EXCOR (BHE) offers circulatory support across all paediatric ages. Clinically, the necessary care and the outcomes differ in various age groups. The EUROMACS database was used to study age- and size-related outcomes for this specific device.

**METHODS:**

All patients <19 years of age from the EUROMACS database supported with a BHE between 2000 and November 2021 were included. Maximally selected rank statistics were used to determine body surface area (BSA) cut-off values. Multivariable Cox proportional hazard regression using ridge penalization was performed to identify factors associated with outcomes.

**RESULTS:**

In total, 303 patients were included [mean age: 2.0 years (interquartile range: 0.6–8.0, males: 48.5%)]. Age and BSA were not significantly associated with mortality (*n* = 74, *P* = 0.684, *P* = 0.679). Factors associated with a transplant (*n* = 175) were age (hazard ratio 1.07, *P* = 0.006) and aetiology other than congenital heart disease (hazard ratio 1.46, *P* = 0.020). Recovery rates (*n* = 42) were highest in patients with a BSA of <0.53 m^2^ (21.8% vs 4.3–7.6% at 1 year, *P* = 0.00534). Patients with a BSA of ≥0.73 m^2^ had a lower risk of early pump thrombosis but a higher risk of early bleeding compared to children with a BSA of <0.73 m^2^.

**CONCLUSIONS:**

Mortality rates in Berlin Heart-supported patients cannot be predicted by age or BSA. Recovery rates are remarkably high in the smallest patient category (BSA <0.53 m^2^). This underscores that the BHE is a viable therapeutic option, even for the smallest and youngest patients.

**Figure:**
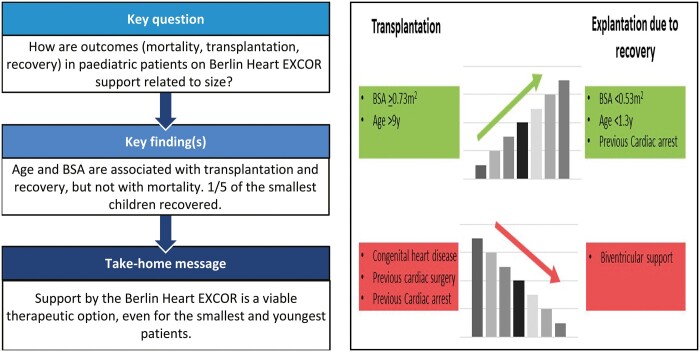

## INTRODUCTION

Around one-third of the paediatric patients awaiting a cardiac transplant weigh less than 10 kg [1]. This subpopulation has higher mortality rates and a greater chance of being delisted because of clinical deterioration compared to larger children [1–3]. Explanations in the literature include different aetiology, e.g. more often congenital heart diseases (CHDs), lower reserve capacity and scarcity of donor hearts for small children [2, 4].

Ventricular assist devices (VAD) have lowered waiting list mortality among children during the last 2 decades [1, 5]. However, various studies have showed inferior outcomes in smaller patients on VAD support compared to larger children. At 1 year after the implant, survival rates around 50–65% are reported in children <1 year compared to 70–85% in older children [4, 6]. In this regard, body surface area (BSA) is reported to have a major effect on the outcomes of children on VAD support [4]. However, within these study populations, a wide heterogeneity exists, with different devices and types of supports (intra- or paracorporeally) taken together. In the smallest children, however, intracorporeal VAD support is not possible, and fewer types of VADs are available.

One of the VAD therapies that is available for small children is the Berlin Heart EXCOR (BHE) VAD (Berlin Heart GmbH, Berlin, Germany). This mechanical, pulsatile pump can be used for short- and long-term support and is placed paracorporeally. The pump chamber is available in 10, 15, 25, 30, 50 and 60 ml to match a patient’s BSA. The 10-ml pump chamber, for example, is meant for patients with a BSA ranging from 0.2 to 0.33 m^2^ [7]. The BHE that has been available since the 1990s can be used to support either the left, the right or both ventricles.

Because outcomes in VAD-supported paediatric patients are worse for smaller children [4], it has been speculated that outcomes in small children on BHE support are poor too. However, the few studies done with subanalyses for smaller patients on BHE support are limited in size and applied different definitions of ‘small children’ (age <1 year, BSA <0.7 or 1.2 m^2^ or weight <5 or <10 kg) [8–12]. These differences hamper the interpretation of the results as well as the ability to draw strong conclusions that would be applicable to daily practice.

The primary goal of this study was to investigate age and BSA as parameters for the outcome after a BHE is implanted and which cut-off values provide the best-fitting results. With this cut-off value, differences in outcomes (survival, transplant, VAD explantation due to recovery) were examined. Second, differences in adverse event rates between the subgroups were studied.

## METHODS

### Data source and patients

**Figure 1:**
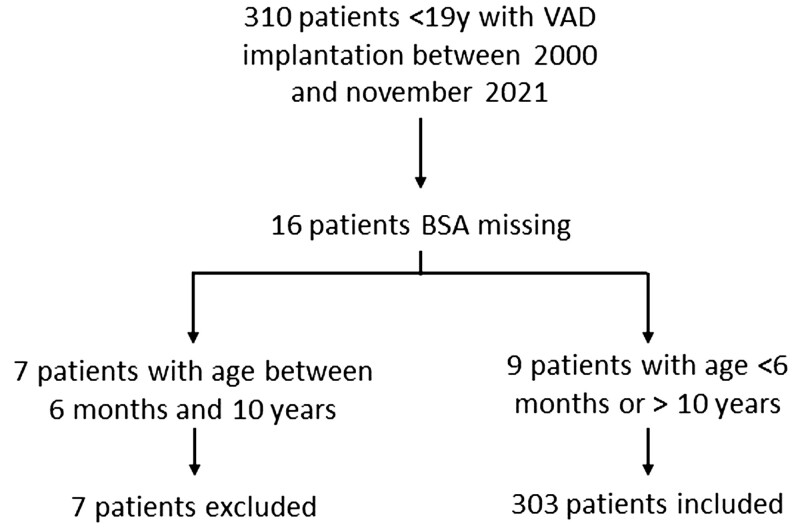
Flow chart of excluded and included patients.

All paediatric patients <19 years of age from the EUROMACS database supported with a BHE between 2000 and November 2021 were included. The available data were studied retrospectively. The study population was divided into subgroups according to BSA. If the BSA of a patient was missing, patients were categorized as ‘BSA <0.53 m^2^’ if they were younger than 6 months and as ‘BSA ≥0.73 m^2^’ if they were older than 10 years of age (Fig. 1) [13]. Because children with a missing BSA, who were between 6 months and 10 years old cannot be divided into one of the BSA groups with absolute certainty, these patients were excluded (Fig. 1). Most centres used the Edmonton protocol as a coagulation protocol. All individual hospitals received approval from their medical or research ethics committee in accordance with *European Journal of Cardio-thoracic Surgery* policy.

### Outcomes

Primary outcomes included mortality, transplant and explant due to recovery, which were considered competing outcomes. Secondary outcomes included early major adverse events: cerebrovascular accident (CVA), pump thrombosis, major bleeding and major infection. Adverse events were reported according to the definitions of the Interagency Registry for Mechanically Assisted Circulatory Support (INTERMACS). Early adverse events were defined as occurring within 30 days after the VAD was implanted.

### Cut points

In this study, cut points for BSA and age were not defined a priori, but a data-driven approach was utilized. For both variables, the maximally selected standardized log-rank statistic for cut points was between the 10% and the 90% quantile of age/BSA. The *P*-value was approximated on an improved Bonferroni inequality [13]. Groups were divided on the cut points of BSA, assuming BSA better represents the size of a patient.

### Statistical analyses

Perioperative characteristics and early outcomes were stratified by BSA category. Categorical data are presented as frequencies (percentage). Continuous data are presented as median (interquartile range). To compare continuous variables, the Student *t*-test (Gaussian distribution) or the Mann–Whitney test (non-Gaussian distribution) was used. The χ^2^ test or Fisher’s exact test (<5 observations per cell) was used to compare categorical variables. A cumulative incidence curve was computed for mortality, transplant and recovery as competing risks using Fine–Gray models and stratified to BSA groups. Cause-specific risk factors for mortality, transplant and recovery were explored using univariable Cox proportional hazard models. Complete case analyses in the univariable modelling were performed because the rates of missing data for the examined parameters were <10% (Supplementary Material, Table S1). Multiple imputation of baseline variables (missingness <30%) using chained equations was performed as sensitivity analyses generating 5 data sets using 20 iterations. Analyses were done on each data set separately and pooled according to Rubins’ rules [14]. Multivariable Cox regression using ridge penalization was performed to explore confounding. Ridge penalization shrinks the coefficient of unimportant variables close to zero and was used to balance the trade-off between relatively few outcomes and a relatively high number of potential covariates [15]. Predictors were not scaled to have unit variance.

### Statistical program

All analyses were performed using International Business Machines Corporation Statistical Package for the Social Sciences (IBM SPSS, Armonk, NY, USA) statistics (version 24) or R (Version 4.0.3) with the packages ‘Survival’, ‘cmprsk’, ‘maxstat’ and ‘Mice’.

## RESULTS

**Figure 2:**
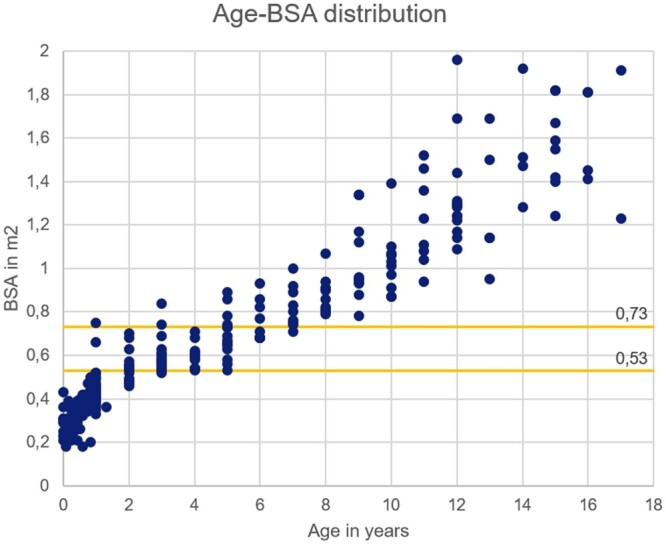
Age–body surface area distribution in the study population. BSA: body surface area.

Originally, 310 patients were included. BSA data were missing for 16 patients (5.2%). Nine patients could still be categorized as either low (<0.53 m^2^) or high (≥0.73 m^2^) BSA based on age. Therefore, only 7 patients were excluded, and a total of 303 patients were included (Fig. 1). Figure 2 depicts the age–BSA distribution in the study population; 48.5% of the patients were male and 65.0% had a primary diagnosis of dilated cardiomyopathy. More than two-thirds (73.0%) were classified as INTERMACS class I or II and in 74.6% only the left ventricle was supported (Table 1).

**Table 1:** Basic characteristics of paediatric patients supported by a Berlin Heart EXCOR per body surface area group

	All (*n* = 303)	BSA <0.53 (*n* = 151)	BSA 0.53–0.72 (*n* = 54)	BSA ≥0.73 (*n* = 98)	*P*-Value
Male sex, n (%)	147 (48.5)	66 (43.7)	34 (63.0)	47 (48.0)	0.052
Age (years), median (IQR)	2.0 (0.6–8.0)	0.6 (0.3–1.0)	3.5 (3.0–5.0)	10.0 (8.0–13.0)	<0.001
BSA (m^2^), median (IQR)	0.5 (0.4–0.9)	0.4 (0.3–0.4)	0.6 (0.5–0.7)	1.1 (0.9–1.4)	<0.001
Primary diagnosis					<0.001
CHD, n (%)	54 (17.8)	29 (19.2)	15 (27.8)	10 (10.2)	
DCM, n (%)	197 (65.0)	104 (68.9)	25 (46.3)	68 (69.4)	
RCM, n (%)	24 (7.9)	2 (1.3)	8 (14.8)	14 (14.3)	
Other,^b^ n (%)	7 (2.3)	1 (0.7)	3 (5.6)	3 (3.1)	
Unknown, n (%)	21 (6.9)	15 (9.9)	3 (5.6)	3 (3.1)	
Creatinine, median (IQR)	44 (33–61)	39 (24–53)	40 (33–61)	59.0 (44–77)	0.025
Albumin, median (IQR)	551 (435–652)	507 (391–620)	575 (442–674)	608.6 (489–688)	0.049
NT-pro-BNP, median (IQR)	21 360 (8681–35 000)	35 000 (17 600–48 660)	14 148 (8770–33 244)	14 999 (7474–30 873)	0.140
INTERMACS classification					0.199
I, n (%)	75 (24.8)	45 (29.8)	12 (22.2)	18 (18.4)	
II, n (%)	146 (48.2)	68 (45.0)	23 (42.6)	55 (56.1)	
III–V, n (%)	57 (18.8)	23 (15.2)	14 (25.9)	20 (20.4)	
Unknown, n (%)	25 (8.3)	15 (9.9)	5 (9.3)	5 (5.1)	
Previous intubation, n (%)	124 (40.9)	80 (53.0)	19 (35.2)	25 (25.5)	<0.001
Previous dialysis, n (%)	9 (3.0)	6 (4.0)	0	3 (3.1)	0.314
Previous ECMO, n (%)	65 (21.5)	31 (20.5)	17 (31.5)	17 (17.3)	0.096
Previous cardiac surgery, n (%)	41 (13.5)	25 (16.6)	11 (20.4)	5 (5.1)	0.007
Previous cardiac arrest, n (%)	41 (13.5)	24 (15.9)	8 (14.8)	9 (9.2)	0.207
Device strategy					0.060
(Possible) bridge to transplant, n (%)	259 (85.5)	123 (81.5)	49 (90.7)	87 (88.8)	
Bridge to recovery, n (%)	26 (8.6)	18 (11.9)	1 (1.9)	7 (7.1)	
Unknown	18 (5.9)	10 (6.6)	4 (7.4)	4 (4.1)	
Type of support					0.004
LVAD, n (%)	226 (74.6)	126 (83.4)	37 (68.5)	63 (64.3)	
BiVAD, n (%)	69 (22.8)	20 (13.2)	16 (29.6)	33 (33.7)	
LVAD + RVAD,^c^ n (%)	7 (2.3)	5 (3.3)	1 (1.9)	1 (1.0)	
RVAD, n (%)	1 (0.3)	0	0	1 (1.0)	
Median time of support, days (IQR)	108 (37–236)	103 (37–194)	220 (46–358)	90 (29–213)	0.207

a The unknown group was not included in the analyses.

b Other = valvular heart disease in 4 patients, hypertrophic cardiomyopathy in 2 patients and cancer in 1 patient.

c When the RVAD was placed during a second operation.

BiVAD: biventricular assist device; BSA: body surface area; CHD: congenital heart disease; DCM: dilated cardiomyopathy; ECMO: extracorporeal membrane oxygenation; INTERMACS: Interagency Registry for Mechanically Assisted Circulatory Support; IQR: interquartile range; LVAD: left ventricular assist device; RCM: restrictive cardiomyopathy; RVAD: right ventricular assist device.

**Table 2:** Multivariable Cox regression models using ridge penalization for mortality, transplant and recovery (pooled results from 5 imputed data sets)

	Mortality		Transplant		Recovery	
	Hazard ratio (95% CI)	*P*-Value	Hazard ratio (95% CI)	P-Value	Hazard ratio (95% CI)	*P*-Value
Age	1.026 (0.957–1.1)	0.472	1.07 (1.021–1.122)	0.006	0.897 (0.796–1.011)	0.086
Male sex	0.859 (0.595–1.239)	0.419	0.96 (0.749–1.232)	0.75	0.739 (0.452–1.208)	0.238
BSA	0.959 (0.545–1.688)	0.886	0.936 (0.625–1.403)	0.75	0.991 (0.457–2.149)	0.982
Primary diagnosis: non–CHD versus CHD	0.845 (0.552–1.294)	0.442	1.467 (1.067–2.018)	0.020	0.919 (0.525–1.611)	0.771
Device strategy: bridge to recovery versus bridge to transplant	0.952 (0.576–1.572)	0.848	0.815 (0.559–1.189)	0.29	3.763 (2.179–6.498)	>0.001
INTERMACS classification (per 1 class)	0.885 (0.638–1.228)	0.469	0.925 (0.742–1.154)	0.491	0.769 (0.499–1.186)	0.245
Intubation	1.184 (0.792–1.769)	0.413	1.217 (0.92–1.608)	0.171	1.094 (0.652–1.834)	0.737
Dialysis	1.124 (0.647–1.952)	0.680	1.183 (0.782–1.79)	0.427	0.996 (0.475–2.089)	0.992
Previous ECMO	1.124 (0.724–1.746)	0.604	0.924 (0.686–1.245)	0.606	0.908 (0.523–1.577)	0.736
Previous cardiac surgery	1.093 (0.707–1.691)	0.690	0.75 (0.543–1.035)	0.082	0.95 (0.524–1.724)	0.868
Previous cardiac arrest	1.141 (0.738–1.762)	0.555	0.795 (0.574–1.102)	0.171	1.376 (0.782–2.422)	0.279
Type of support: biventricular versus univentricular	0.763 (0.519–1.122)	0.174	1.067 (0.813–1.399)	0.641	1.733 (0.97–3.098)	0.074

BSA: body surface area; CHD: congenital heart disease; CI: confidence interval; ECMO: extracorporeal membrane oxygenation; INTERMACS: Interagency Registry for Mechanically Assisted Circulatory Support.

### Cut points

**Figure 3:**
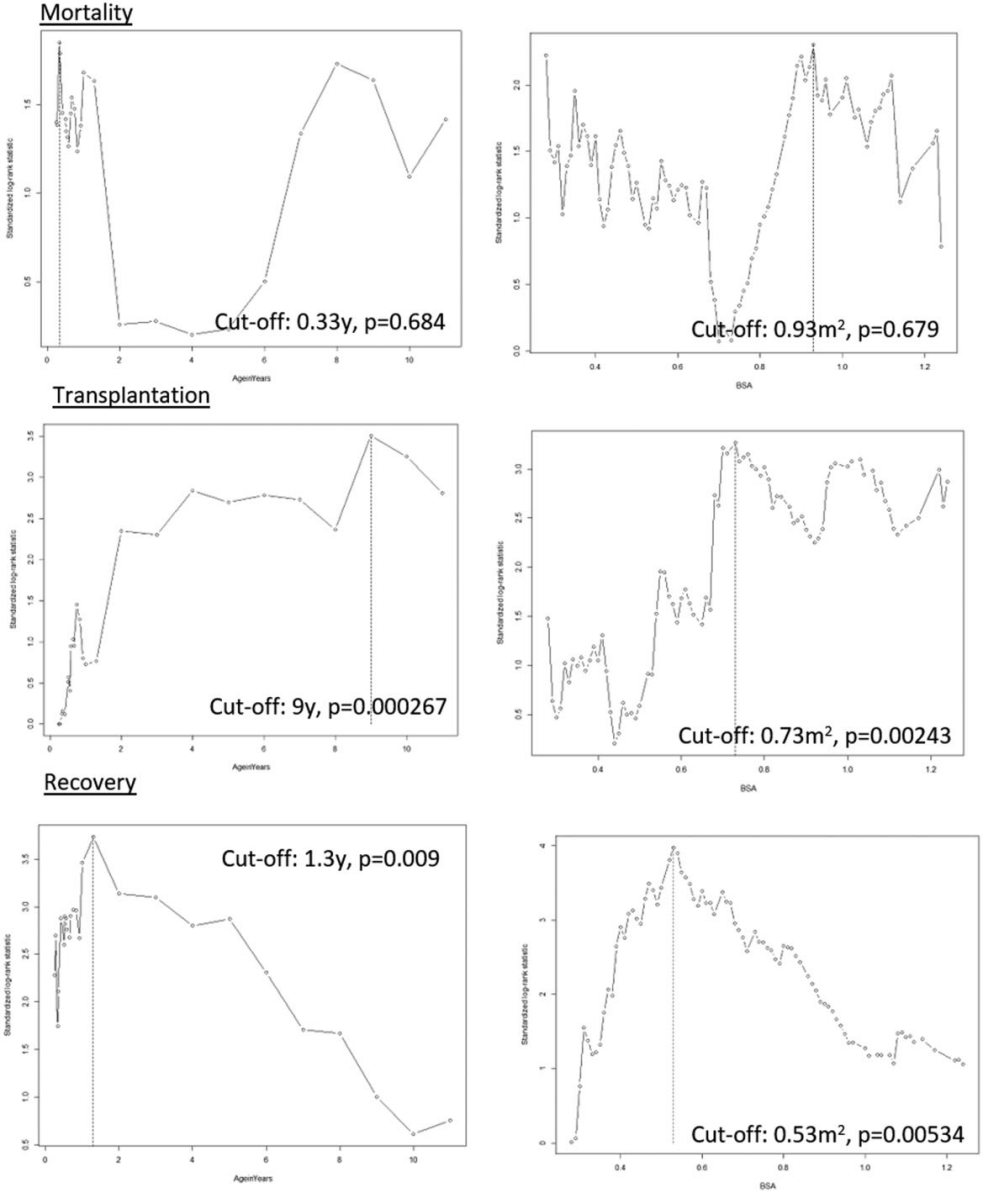
Maximally selected rank statistics: predictive value of age and body surface on outcomes [mortality (**a** and **b**), transplant (**c** and **d**), recovery (**e** and **f**)]. BSA: body surface area.

At which BSA or age cut-off the log-rank statistic was the highest (and therefore most likely to be significant) for a certain outcome was determined empirically. A significant cut-off for BSA or age was not found for death. The lowest *P*-values were obtained for an age of 0.33 years (*P* = 0.684) and a BSA of 0.93 m^2^ (*P* = 0.679) (Fig. 3). A significant age and BSA cut-off value for a transplant and recovery as outcomes were identified. Patients above 9 years of age and patients with a BSA of ≥0.73 m^2^ received a transplant significantly more often (*P* = 0.000267 and *P* = 0.00243) (Fig. 3). Furthermore, in patients <1.3 years of age and patients with a BSA of <0.53 m^2^, a BHE was explanted significantly more often due to recovery (*P* = 0.009 and *P* = 0.00534). Based on these analyses, BSA cut points of <0.53 (*n* = 151), 0.53–0.72 (*n* = 54) and BSA ≥0.73 (*n* = 98) were used in this study.

### Baseline characteristics

**Figure 4:**
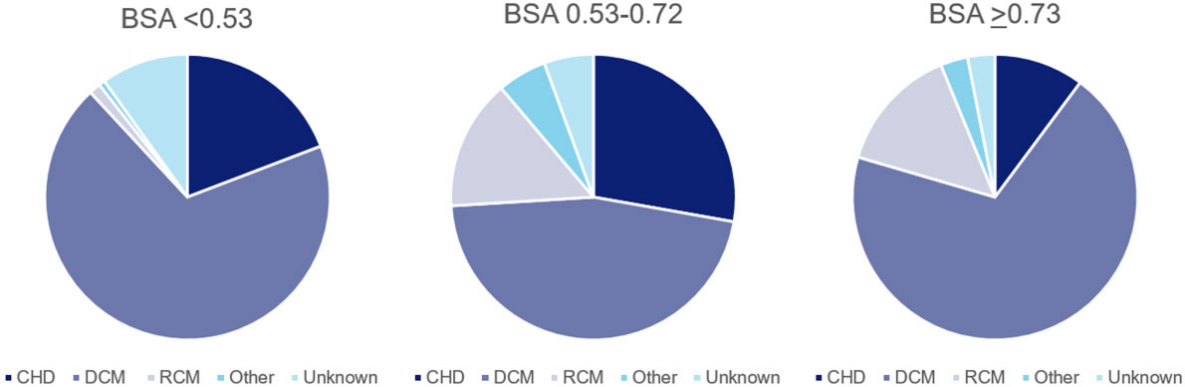
Distribution of aetiology in the different body surface area groups. **Other = valvular heart disease in 4 patients, hypertrophic cardiomyopathy in 2 patients and cancer in 1 patient. BSA: body surface area; CHD: congenital heart disease; DCM: dilated cardiomyopathy; RCM: restrictive cardiomyopathy.

Aetiology differed significantly between the BSA groups, with higher percentages of CHD in the first 2 groups (19.2–27.8 vs 10.2%, *P* < 0.001) and the lowest percentage of dilated cardiomyopathy in group 2 (46.3 vs 68.9–69.4%, *P* < 0.001) (Table 1). The differences in aetiology between the BSA groups are also depicted in Fig. 4. Furthermore, smaller patients were more often intubated (53.0 vs 35.2 vs 25.5%, *P* < 0.001), and patients with a BSA of <0.73 m^2^ more often had a previous cardiac operation (16.6–20.4 vs 5.1%, *P* = 0.007). A biventricular assist device (BiVAD) was more common in larger children (29.6–33.7 vs 13.2%, *P* = 0.004) (Table 1). Creatinine and albumin levels also differed significantly between the BSA groups; unfortunately, many data regarding these laboratory values were missing (Tables 1 and 3 and Supplemental Material, Table S1).

**Table 3:** Early adverse events per body surface area group

	All (*n* = 303)	BSA <0.53 (*n* = 151)	BSA 0.53–0.72 (*n* = 54)	BSA ≥0.73 (*n* = 98)	*P*-Value
Patients with early CVA, n (%)	30 (9.9)	15 (9.9)	6 (11.1)	9 (9.2)	0.929
Total episodes of early CVA	32	15	7	10	
Contributed to death, n (%)	11 (36.7)	5 (33.3)	2 (33.3)	4 (44.4)	0.846
Patients with early pump thrombosis, n (%)	52 (17.2)	30 (19.9)	12 (22.2)	10 (10.2)	0.079
Total episodes of early pump thrombosis	78	44	17	17	
Patients with early major bleeding, n (%)	40 (13.2)	17 (11.3)	4 (7.4)	19 (19.4)	0.069
Total episodes of early major bleeding	57	21	7	29	
Patients with early major infection, n (%)	27 (8.9)	13 (8.6)	7 (13.0)	7 (7.1)	0.476
Total episodes of early major infection	28	14	7	7	

BSA: body surface area; CVA: cerebrovascular accident.

### Competing primary outcomes

**Figure 5:**
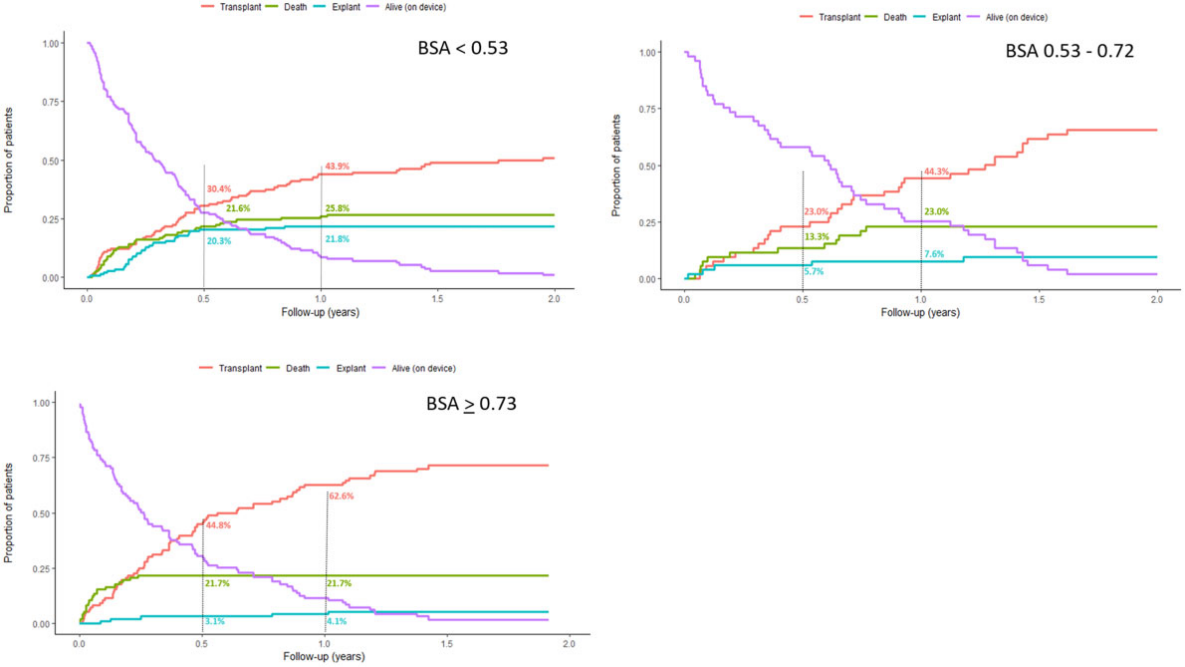
Competing outcomes per body surface area group. BSA: body surface area.

Figure 5 depicts the competing outcomes per BSA group. A difference in the distribution of outcomes at 6 months and 1 year is visible. Transplant rates were higher in patients with a BSA of ≥0.73 m^2^ at 6 months (44.8% vs 23.0–30.4%) and at 1 year (62.6% vs 43.9–44.3%). In the smallest patients (BSA <0.53 m^2^), recovery rates were remarkably higher (21.8% vs 4.1–7.6%). Mortality rates were similar between the different BSA groups, especially at 1 year after a VAD implant. Another remarkable finding is the difference in time on support, which seemed higher in the second BSA group (BSA 0.53–0.72) compared to the other 2 BSA groups (220 days vs 103 and 90 days, *P* = 0.207) (Table 1). Although this finding was not significantly different, it was also reflected by data shown in Fig. 5: At the 1-year follow-up, 58% of patients in the 0.53–0.72 BSA groups are still on device therapy, whereas 27.7% and 30.4% in the <0.52 group and >0.73 group are still on device therapy.

### Predictors of primary outcomes

In univariable Cox proportional hazard regression, age and BSA were found to be predictive of receiving a transplant and recovery but not of death (Supplementary Material, Table S2). Hazard ratios of the multivariable Cox regression for mortality, transplant and recovery are shown in Table 2. BSA and age were not associated with mortality or recovery in multivariable analyses, but higher age was significantly associated with transplant [hazard ratio (HR): 1.07, *P* = 0.006]. In addition, paediatric patients with a primary diagnosis of cardiomyopathy or non-congenital valvular heart disease (compared to CHD) were more likely to receive transplants (HR: 1.5, *P* = 0.020). In a post hoc sensitivity analyses without device strategy in the model, none of the other predictors became significant (Supplementary Material, Table S3).

### Early adverse events

When the 3 BSA groups were compared, early adverse events (within 30 days after the VAD was implanted) did not occur significantly more often in one of the groups, but a trend for pump thrombosis and early bleeding was seen (Table 3). When collapsing the <0.53 and 0.53–0.72 BSA groups into 1 group versus all the children with a BSA of >0.73 m^2^ in a post hoc analysis, significantly fewer patients with early pump thrombosis were seen (20.5 vs 10.2%, *P* = 0.026) and significantly more early major bleeding events (10.2 vs 19.4%, *P* = 0.028) in the larger children were noted.

## DISCUSSION

### Mortality

This study showed that age and BSA are not predictive of mortality in the BHE supported population (Table 2 and Fig. 3). This finding contrasts with findings in some of the earlier published literature. For example, a previously published North American study [16] of 73 paediatric patients supported by a BHE found BSA (HR 0.16, 95% CI 0.04–0.91 *P* = 0.03) in univariable analyses and age at implant in both univariable (HR 0.53, 95% CI 0.36–0.76, *P* = 0.00006) and multivariable analyses (HR 0.59, 95% CI 0.46–0.78, *P* < 0.0001) to be a significant predictor of death [16]. Their study population, however, differed greatly from ours: 97% had an INTERMACS classification of I or II; 42.5% of patients were supported by a BiVAD, and patients were included between 2000 and 2007. BiVAD support was also found to be a significant predictor of mortality (HR 4.61, 95% CI 1.67–12.7, *P* = 0.003) [16]. Another study found that patients with a body weight of <10 kg had a significantly worse survival (*P* < 0.0001) [8]. A body weight of 10 kg is comparative to a BSA of ∼0.45–0.48 m^2^ [17]. This population, however, had a worse clinical state when the VAD was implanted (52.5% INTERMACS I), and more children <10 kg were supported by extracorporeal membrane oxygenation prior to VAD support (48.5% vs 33.6%). Furthermore, this study only enrolled patients between 2007 and 2010 [8]. The aforementioned 2 studies might indicate a possible learning curve and improved patient selection for supporting small children: The size of a child was predictive of death in the BHE-supported population but is currently no longer a risk factor.

The Paedi-EUROMACS report showed an overall significantly worse survival in small children (<1 year, BSA <1.0 m^2^ and weight <5 kg) [4]. Even within the paracorporeal supported VAD population, a lower body weight (<5 kg) was associated with significantly lower survival (51% vs 70% and 87% at 6 months, *P* = 0.0022). This group is, however, still a group with different types of devices [4]. In this study, INTERMACS classification, previous cardiac surgery and type of support were similar to those factors in our study, not predictive factors of mortality (Table 2).

### Transplant

Transplant rates, on the other hand, can be predicted by age and BSA in the BHE-supported population, with larger children (>9 years or BSA ≥0.73 m^2^) having better chances of receiving a donor heart (Figs. 3 and 5). This finding could be explained by the greater availability of donor hearts in larger children [3]. Patients with CHD were less likely to receive a transplant (Table 2). Most patients who have a CHD and are on the transplant waiting list have had 1 or more operations, leaving them with an altered and adapted circulatory system with adhesions. Furthermore, long-standing irreversible pulmonary hypertension is more often present in these children, making them less appealing for a transplant. However, reduced transplant rates in paediatric patients with CHD can probably be explained primarily by the larger numbers of deaths among those on the waiting list in this subpopulation [1, 2].

### Recovery

One of the most interesting findings of this study was the higher recovery rate in children with a BSA of <0.53 m^2^ (21.2% vs 6.6%, *P* = 0.00534) (Fig. 3). In one-fifth of these small children, the VAD could be explanted because of the restoration of cardiac function, postponing or even completely avoiding the need for a transplant. Given the scarcity of donor hearts, especially in infants, this is an impressive outcome, making VAD therapy in the smallest children more appealing.

### Adverse events

Results from earlier published studies of small cohorts suggest a difference in CVA or brain injury rates in small versus larger children [9, 18]. Contrarily, our study showed that the incidence of CVA was comparable in the different BSA groups (*P* = 0.929). Children with a larger BSA (≥0.73 m^2^), however, had significantly fewer episodes of early pump thrombosis (20.5 vs 10.2%, *P* = 0.026) and significantly more episodes of early major bleeding (10.2 vs 19.4%, *P* = 0.028). This shift from thrombotic complications to haemorrhagic complications might reflect the evolving and poorly understood coagulation system in early childhood, whereas the most used anticoagulation protocol for BHE-supported children, the Edmonton protocol, is the same for all patients above 1 year of age [19]. Although many centres deviate from the Edmonton protocol, there is often a dichotomy between children below 6, 8, 12 or 24 months and older paediatric patients [20]. Perhaps the ‘one approach fits all’ protocol should be revised because great differences exist within the paediatric population. Of additional interest regarding BSA and the risk of thrombosis is the fact that a too-large pump compared to body size has also been proven to increase the risk of thromboembolic events [7]. This situation is, obviously, more likely to happen in smaller children. Of note, only univariable analyses are performed, and other underlying factors that differ between the BSA groups may be related to this observation.

### The Future

Paediatric VAD support still has high mortality and adverse event rates and therefore needs to be further improved. Fortunately, exciting new inventions and developments are on the horizon. For example, a new miniaturized device for the smallest children is currently being developed. The Jarvik Paediatric 2015 (Jarvik Heart, Inc., New York, NY, USA) is a continuous flow device that has a diameter of only 15 mm and can be placed intracorporeally [21]. The original design (Jarvik 2000) was even smaller but was rejected by the US Food and Drug Administration due to an increased risk of haemolysis, demonstrated with in vitro testing [21]. At this writing, the first clinical trial (PumpKIN trial) with the Jarvik 2015 is running in the United States and is expected to finish at the end of this year, with included patients between 8 and 20 kg [21], 8 kg being comparable to a BSA between 0.42 and 0.44 m^2^ [22].

In addition, the EXCOR Active driving unit (Berlin Heart) has recently become available. This driving unit weighs only 15 kg and has a battery runtime of 6.5 h. It allows more mobility in BHE-supported children and sometimes even discharge home. It increases the quality of life in these severely sick children who sometimes have to wait for months for a donor heart [23].

Furthermore, the EXCOR Venous Cannula device (Berlin Heart) supports subpulmonary circulation in patients with a failing Fontan circulation. It can restore haemodynamics and therefore support organ recovery [24].

### Limitations

This study includes all paediatric patients from the EUROMACS database supported with a BHE. This study, however, also has some limitations. First and foremost, some data were missing, which is partially inevitable due to the fact that the study was conducted using registry data. The amount of missing data was minimalized by asking the participating centres in advance to fill in the database as completely as possible. Furthermore, we approached some of the centres afterwards to complete the data. In addition, to limit the impact of missing data on the results, parameters for which >10% of the data were missing were not studied with Cox proportional hazard regression analyses, and multiple imputation was used to supplement missing variables in multivariable models. Another limitation of this study was the interobserver bias caused by the multicentre nature of this study. The impact of interobserver bias on the results was minimalized by providing detailed definitions of and criteria for adverse events. The sample size of this cohort remains limited and potentially underpowered to detect differences, especially in the recovery and mortality end points with relatively few events.

## CONCLUSION

BSA and age were not associated with death. Transplant and recovery differ significantly in various BSA categories, but BSA was not a significant predictor of death, transplant or recovery in multivariable analyses. Most noteworthy is the large number of explants due to recovery in children with a BSA of <0.53 m^2^, making the BHE a viable option for smaller children, not only as a bridge to transplant but also as a bridge to recovery.

## Supplementary Material

ezac516_Supplementary_Data

## Data Availability

The data underlying this article are available in the article and in its online supplementary material. All relevant data are within the manuscript and its Supporting Information files.

## Abbreviations

BHE: Berlin Heart EXCOR
BiVAD: biventricular assist device
BSA: body surface area
CHD: congenital heart disease
CVA: cerebrovascular accident
HR: hazard ratio
INTERMACS: Interagency Registry for Mechanically Assisted Circulatory Support
VAD: ventricular assist devices
