# Supplementary material for: Outcomes in small children on Berlin Heart EXCOR support: age and body surface area as clinical predictive factors
Source: Eur J Cardiothorac Surg. 2022 Nov 8;63(1):ezac516. doi: 10.1093/ejcts/ezac516 (PMC9825200; doi:10.1093/ejcts/ezac516)
Supplement: ezac516_Supplementary_Data [file ezac516_supplementary_data.docx]

**Supplemental material**

**Supplementary table 1:** Missing data

|  | All (n=303) | BSA <0.53 (n=151) | BSA 0.53-0.72 (n=54) | BSA >0.73  (n=98) |
| --- | --- | --- | --- | --- |
| BSA | 9* |  |  |  |
| Age | 0 | 0 | 0 | 0 |
| Type of Device (LVAD/BiVAD) | 0 | 0 | 0 | 0 |
| Primary diagnosis | 21 (6.9) | 15 (9.9) | 3 (5.6) | 3 (3.1) |
| Creatinine | 88 (29.0) | 45 (29.8) | 15 (27.8) | 28 (28.6) |
| Albumin | 156 (51.5) | 75 (49.7) | 25 (46.3) | 56 (57.1) |
| NT-pro-BNP | 198 (65.3) | 104 (68.9) | 35 (64.8) | 59 (60.2) |
| INTERMACS classification | 25 (8.3) | 15 (9.9) | 5 (9.3) | 5 (5.1) |
| Previous intubation | 20 (6.6) | 14 (9.3) | 3 (5.6) | 3 (3.1) |
| Previous dialysis | 20 (6.6) | 14 (9.3) | 3 (5.6) | 3 (3.1) |
| Previous ECMO | 28 (9.2) | 17 (11.3) | 5 (9.3) | 6 (6.1) |
| Previous cardiac surgery | 24 (7.9) | 14 (9.3) | 4 (7.4) | 6 (6.1) |
| Previous cardiac arrest | 22 (7.3) | 16 (10.6) | 3 (5.6) | 3 (3.1) |
| Device strategy | 18 (5.9) | 10 (6.6) | 4 (7.4) | 4 (4.1) |
| Primary outcome (lost to follow up) | 4 (1.3) | 2 (1.3) | 1 (1.9) | 1 (1.0) |

*These patients could still be classified as either low (<0.53m^2^) or high (>0.73m^2^) BSA based on age.

BSA = body surface area, ECMO = extracorporeal membrane oxygenation, INTERMACS = Interagency Registry for Mechanically Assisted Circulatory Support

**Supplementary table 2:** Univariable Cox Proportional Hazard Regression models for mortality, transplantation and recovery

|  | **Mortality** | | **Transplantation** | | **Recovery** | |
| --- | --- | --- | --- | --- | --- | --- |
|  | *Hazard ratio (95% CI)* | *P-value* | *Hazard ratio (95% CI)* | *P-value* | *Hazard ratio (95% CI)* | *P-value* |
| Sex | 0.814 (0.513-1.292) | 0.383 | 0.901 (0.667-1.217) | 0.497 | 0.599 (0.321-1.118) | 0.107 |
| Age | 1.011 (0.961-1.064) | 0.676 | 1.063 (1.029-1.099) | <0.001 | 0.872 (0.785-0.968) | 0.010 |
| BSA | 1.151 (0.624-2.124) | 0.653 | 1.816 (1.232-2.677) | 0.003 | 0.144 (0.037-0.570) | 0.006 |
| BSA group | 0.920 (0.703-1.205) | 0.545 | 1.232 (1.033-1.47) | 0.02 | 0.453 (0.284-0.723) | <0.001 |
| Primary diagnosis: non-CHD vs. CHD | 0.691 (0.397-1.204) | 0.192 | 3.311 (1.913-5.729) | <0.001 | 0.679 (0.327-1.41) | 0.299 |
| INTERMACS classification | 0.747 (0.527-1.058) | 0.101 | 0.976 (0.784-1.214) | 0.826 | 0.740 (0.455-1.204) | 0.225 |
| Previous ECMO | 1.557 (0.934-2.596) | 0.090 | 0.782 (0.530-1.156) | 0.218 | 1.009 (0.480-2.12) | 0.981 |
| Previous cardiac surgery | 1.391 (0.773-2.505) | 0.271 | 0.428 (0.257-0.712) | 0.001 | 1.078 (0.474-2.451) | 0.858 |
| Previous cardiac arrest | 1.565 (0.868-2.824) | 0.137 | 0.558 (0.327-0.952) | 0.032 | 2.287 (1.141-4.584) | 0.020 |
| Device strategy: bridge to recovery vs. bridge to transplantation | 0.778 (0.282-2.149) | 0.628 | 0.303 (0.096-0.952) | 0.041 | 10.42 (5.558-19.54) | <0.001 |
| Type of support: biventricular vs. univentricular | 1.605 (0.993-2.59) | 0.053 | 1.024 (0.727-1.443) | 0.891 | 0.226 (0.070-0.730) | 0.013 |

BSA = body surface area, CHD = congenital heart disease, ECMO = extracorporeal membrane oxygenation, INTERMACS = Interagency Registry for Mechanically Assisted Circulatory Support.

**Supplementary table 3**: Sensitivity analyses of multivariable cox proportional hazard regression for recovery with ridge penalization temporarily excluding device strategy: bridge to recovery vs bridge to transplant (results of 5 pooled imputed datasets).

|  | *Hazard ratio (95% CI)* | *P-value* |
| --- | --- | --- |
| Age | 0.894 (0.793-1.008) | 0.078 |
| Male sex | 0.727 (0.446-1.185) | 0.21 |
| BSA | 1.009 (0.465-2.189) | 0.983 |
| Primary diagnosis: non-CHD vs. CHD | 0.865 (0.49-1.525) | 0.619 |
| Device strategy: bridge to recovery vs. bridge to transplantation | 0.799 (0.525-1.216) | 0.303 |
| Intermacs classification _(per 1 class)_ | 1.135 (0.68-1.894) | 0.631 |
| Intubation | 0.982 (0.47-2.053) | 0.962 |
| Dialysis | 0.841 (0.487-1.452) | 0.54 |
| Previous ECMO | 0.965 (0.535-1.74) | 0.907 |
| Previous cardiac surgery | 1.441 (0.82-2.53) | 0.214 |
| Previous cardiac arrest | 1.739 (0.975-3.101) | 0.071 |
| Type of support: biventricular vs. univentricular | 0.894 (0.793-1.008) | 0.078 |

BSA = body surface area, CHD = congenital heart disease, ECMO = extracorporeal membrane oxygenation, INTERMACS = Interagency Registry for Mechanically Assisted Circulatory Support.
